# Herpes Zoster Risk in Patients with Rheumatoid Arthritis and Its Association with Medications Used

**DOI:** 10.3390/ijerph20032123

**Published:** 2023-01-24

**Authors:** Sithembiso Tiyandza Dlamini, Kyaw Moe Htet, Ei Chue Chue Theint, Aerrosa Murenda Mayadilanuari, Wei-Ming Li, Yi-Ching Tung, Hung-Pin Tu

**Affiliations:** 1 Graduate Institute of Medicine, College of Medicine, Kaohsiung Medical University, Kaohsiung 807378, Taiwan; 2 M. Sc. Program in Tropical Medicine, College of Medicine, Kaohsiung Medical University, Kaohsiung 807378, Taiwan; 3 Department of Urology, Kaohsiung Medical University Hospital, Kaohsiung 807378, Taiwan; 4 Department of Urology, School of Medicine, College of Medicine, Kaohsiung Medical University, Kaohsiung 807378, Taiwan; 5 Department of Urology, Ministry of Health and Welfare, Pingtung Hospital, Pingtung 90054, Taiwan; 6 Department of Public Health and Environmental Medicine, School of Medicine, College of Medicine, Kaohsiung Medical University, Kaohsiung 807378, Taiwan; 7 Department of Medical Research, Kaohsiung Medical University Hospital, Kaohsiung 807378, Taiwan

**Keywords:** observational study, incidence rate ratio, rheumatoid arthritis, prednisolone, herpes zoster

## Abstract

Rheumatoid arthritis (RA) was associated with the risk of incident herpes zoster (HZ), which might be influenced by medication use by RA patients. We aimed to investigate the association of RA with the risk of incident HZ and how the HZ risk effected by RA medications in CIC RA patients. We conducted an observational study including population-based representative insurance claims data of 19,673 patients with RA and 39,346 matched patients without RA during 1997–2010 from the Taiwan National Health Insurance Research Database; we identified 1651 patients with catastrophic illness-certified (CIC) RA and 11,557 matched patients with non-CIC RA. Exploratory analyses assessed the association between RA/CIC RA and risk of incident HZ and its complications. The association of prescribed medications with HZ risk in CIC RA patients was also estimated. The incidence rates of HZ were higher in CIC RA patients and non-CIC RA than in the matched people without RA (21.95 and 14.03 vs. 7.36 events per 1000 person-years, respectively). The adjusted incidence rate ratio (95% confidence interval (CI)) for HZ was 1.74 (1.65–1.84) in RA patients vs. matched non-RA and 1.65 (1.44–1.89) in CIC RA patients vs. non-CIC RA. For HZ complications, RA had a 2.85-fold higher risk than non-RA, and CIC RA had a 1.78-fold higher risk than non-CIC RA. Moreover, in CIC RA patients, prednisolone use was associated with incident HZ risk compared with prednisolone nonuse (adjusted odds ratio 1.48, 1.08–2.03); prolonged prednisolone use (approximately 5 years) increased the risk (adjusted odds ratio 2.16, 1.46–3.19). Our results suggested that RA was positively associated with HZ risk, particularly in RA patients with prednisolone use.

## 1. Introduction

Herpes zoster (HZ) is a neurocutaneous disease caused by the reactivation of latent varicella-zoster virus (VZV) [1]. Early treatment is the best method to improve the quality of life and prevent postherpetic complications. VZV belongs to the human alpha-herpesviruses and can cause two disease manifestations: chickenpox (i.e., varicella) and shingles (i.e., HZ) [1]. Chickenpox is a common contagious childhood disease characterized by itchy blisters, but it rarely causes serious problems [2]. VZV persists in the sensory ganglia of the cranial nerves and in the spinal dorsal-root ganglia after varicella resolves and can become reactivated after many decades of latency [1,2]. HZ may have the direct involvement of the ganglia and the destruction of neurons during VZV reactivation [2]. Complications of HZ include bacterial superinfection, ophthalmic complications, meningitis and nervous-system complications [3,4,5]. Specific T-cell immunity is responsible for the body’s defense against VZV reactivation [6]. Declining cellular immunity, due to increasing age or immunosuppression, triggers VZV reactivation [7]. Thus, HZ occurs more frequently in patients with malignancies, human immunodeficiency virus (HIV) infection, transplantation, and immunosuppressive disorders; and in those receiving immune suppressants [7,8]. 

Rheumatoid arthritis (RA) is a chronic systemic inflammatory disease that causes inflammation of the small joints of the hands and feet, causing painful, swollen, and even fused joints that can cause lifelong disability [9]. In Taiwan, the National Health Insurance program had defined many categories of serious illnesses or injuries as “catastrophic illnesses”. Patients have to undergo a rigorous regulatory review before obtaining a Catastrophic Illness Certificate (CIC) for RA. A patient with CIC RA can receive free medical care (outpatient or inpatient care) and other support from the Taiwanese government [10]. 

Patients with rheumatoid arthritis (RA) have many comorbidities, some of which might be due to the disease itself; however, most of them are due to the adverse effects of medications for RA—infections being the most common adverse consequences [11,12]. Among the infections, tuberculosis and HZ have been frequently encountered [12]. The effect of tuberculosis on patients with RA has been a substantial topic of research over the past decade [13,14]. However, HZ has not been discussed adequately thus far [15,16,17,18]. After Jak kinase inhibitors were introduced to the treatment of patients with RA, HZ drew considerable attention [19,20,21,22]. HZ might have a negative influence on the quality of life of RA patients [23,24]. The acute complications of HZ include moderate to severe pains, which can increase both physical and mental stress, especially in the elderly people with lower health utility values and worse health status [25,26]. Chronic HZ will also lead to chronic pains and nervous-system complications [27].

On literature reviews of the HZ risk in RA patients, it was found to be influenced by the medications that are used to treat RA. In Japan, the HZ incidence in patients was higher in RA patients than the general population (9.1 vs. 4.15 per 1000 person-years, respectively) [28]. In the United States, the HZ incidence in RA patients was 12.1 cases per 1000 person-years, compared with 5.4 cases per 1000 person-years for controls [17].The main groups of medications include steroids, disease-modifying antirheumatic drugs (DMARDs), and biopharmaceutical agents. Prednisolone, a strong anti-inflammatory agent, was observed to increase the risk of HZ during RA treatment, especially with a high dosage (1 mg/day increment—1.13 fold [29]; >10 mg/day use—2.3 fold) [30]. Methotrexate, the most commonly used synthetic DMARD, also caused a 1.58-fold increase in the HZ risk [30]. Tissue necrosis factor inhibitors (TNFi) and biopharmaceutical agents caused a 1.88-fold increase in the HZ risk [31]. Herpes zoster infections occurred more with JAKi use by 3.66 times than with DMARD use, and 1.9–2.3 times more than with the use of biopharmaceutical agents (JAKi is newer and effective for the treatment of RA) [32]. However, the association of combined medications in Asian patients is still uncertain, and little is known about outcomes of HZ risk in patients with RA after treatment with combined medications.

In this study, by using the Taiwan National Health Insurance (NHI) Research Database (NHIRD), we investigated whether patients with RA might have a higher risk of getting HZ—and the same for CIC RA—and their risk of getting HZ complications. Additionally, the use of frequently prescribed medications and combined medications’ effects on the risk of HZ infections in CIC RA patients were also explored. 

## 2. Materials and Methods

### 2.1. Source of Data and Study Population

In this retrospective cohort study, the NHIRD was used. This dataset represents a nationally representative group of 1 million individuals randomly selected from among all individuals insured under Taiwan NHI. In the NHIRD, diagnosis coding is performed according to the International Classification of Diseases, Ninth Revision, Clinical Modification (ICD-9-CM) diagnostic criteria. The study protocol was reviewed and approved by the Institutional Review Committee of Kaohsiung Medical University Hospital, Taiwan (KMUHIRB-EXEMPT (I)-20190011).

### 2.2. Inclusion and Exclusion Criteria 

**Figure 1:**
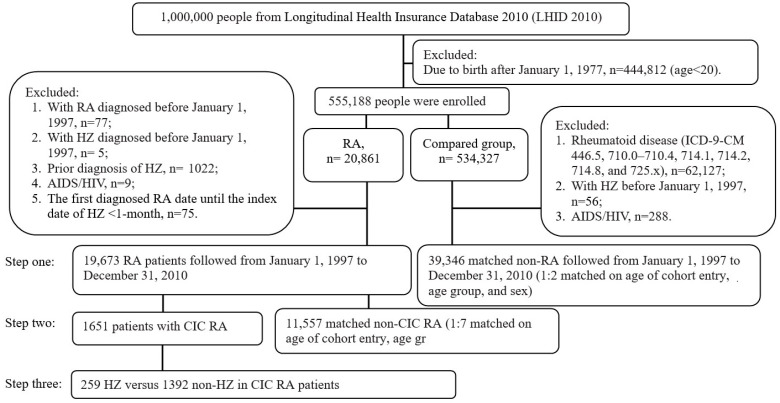
Flowchart for the selection of patients. RA: rheumatoid arthritis; CIC: catastrophic illness-certified; HZ: herpes zoster; comparison group = non-RA.

We included patients aged ≥20 years who received a new diagnosis of HZ. The patients were followed for a maximum of 14 years. For patients with RA, the exclusion criteria were as follows: RA or HZ diagnosed before January 1, 1997; prior diagnosis of HZ or AIDS/HIV; and time between the date of first diagnosis of RA to index date of HZ < 1 month. In the compared group (non-RA), the exclusion criteria were as follows: rheumatoid disease (ICD-9-CM 446.5, 710.0–710.4, 714.1, 714.2, 714.8, and 725.x), HZ diagnosed before 1 January 1997, and prior diagnosis of AIDS/HIV (Figure 1).

### 2.3. Ascertainment of RA and CIC RA

Non-RA patients (compared group) were selected from a random sample of the ambulatory care data file of the 2010 Longitudinal Health Insurance Database (LHID2100) with the inclusion period of 1 January 1997 to 31 December 2010. The primary case definition of RA was a physician-recorded primary diagnosis (ICD-9-CM 714.0) at an outpatient or inpatient visit. In step one, 19,673 patients with RA and 39,346 matched patients without RA (1:2 ratio) were identified. However, this study is based on claims data, so there is always the inherent information bias; hence, to evaluate the robustness of case assessment, we stratified patients based on whether patient had catastrophic illness-certified (CIC) RA with prescribed medication use. In Taiwan, patients with RA can apply for catastrophic illness certification to be exempted from copayments for healthcare costs related to RA [33,34]. Patients with CIC RA are required to have thorough clinical and laboratory evaluations, fulfillment of appropriate classification criteria, and review by a physician commissioned by the NHI Bureau, and thus, their data are highly accurate and reliable [33]. Once one is evaluated as having CIC RA, the status is life long, and there is no need for further evaluations. In step two, 1651 RA patients with CIC RA and 11,557 matched patients with non-CIC RA (1:7 ratio) were included. In step three, the associations of prescribed medications and HZ were estimated among 1651 patients with CIC RA.

### 2.4. Medications Used in RA

The following medications were included. (1) Corticosteroid agents were prednisolone (Anatomical Therapeutic Chemical code H02AB06), methylprednisolone (H02AB04), and dexamethasone (H02AB02). (2) Biopharmaceutical agents were (i) anti-tumor necrosis factor α (anti-TNF-α) agents, including etanercept (L04AB01) and adalimumab (L04AB04); and (ii) a B-cell-depleting agent, rituximab (L01XC02). (3) Disease-modifying antirheumatic drugs (DMARDs) were azathioprine (L04AX01), methotrexate (MTX, L01BA01), sulfasalazine (A07EC01), hydroxychloroquine (P01BA02), leflunomide (L04AA13), and cyclosporin (L04AA01). (4) Other drugs were cyclophosphamide (L01AA01) and penicillamine (M01CC01). Before 2010, biopharmaceuticals abatacept and tocilizumab were unavailable in Taiwan. 

### 2.5. HZ Outcomes Assessment

HZ events were defined as new HZ events (ICD-9-CM 053.x—including phenotypes of HZ with meningitis, 053.0; HZ with nervous-system complications, 053.1; and HZ with ophthalmic complications, 053.2) identified at an outpatient or inpatient visit.

### 2.6. Comorbidities

In addition to the demographic risk factors of age, sex, and region, we evaluated other potentially confounding factors: lipid metabolism disorders, obesity, alcohol abuse, hypertension, myocardial infarction, congestive heart failure, peripheral vascular diseases, cerebrovascular diseases, dementia, chronic pulmonary disease, peptic ulcer disease, mild liver disease, diabetes without chronic complications, diabetes with chronic complications, hemiplegia or paraplegia, renal diseases, any malignancy (including leukemia and lymphoma and excluding malignant neoplasms of skin), moderate or severe liver disease, and metastatic solid tumor—all conditions diagnosed according to ICD-9-CM [34].

### 2.7. Statistical Analysis

Propensity score-matched analysis was performed on the association between RA and non-RA and CIC RA and non-CIC RA. Continuous and categorical variables were analyzed using t or Wilcoxon rank sum tests and chi-squared tests, respectively; the values obtained for the RA and matched compared group were compared. The incident rate ratios (IRRs) were calculated using the generalized log-linear model and by performing Poisson regression analysis. For follow-up analysis, we calculated RA and incident HZ risk at 1-year follow-up. Potential risk factors, such as comorbidities, were incorporated into the model. Interactions between RA and sex were tested using the generalized linear model with an added interaction term and potential risk factors. The associations of patients with CIC RA and prescribed medications and HZ were estimated, and adjusted odds ratios (ORs) were calculated after adjustments for covariates by using a multiple logistic regression model. All statistical analyses were performed using SAS (version 9.4, SAS Institute, Cary, NC, USA).

## 3. Results

For step one, Table 1 presents the demographic characteristics of the study population. We identified 19,673 RA cases (mean age: 46.2 ± 13.6 years) and 39,346 matched non-RA group individuals (mean age: 46.0 ± 13.6 years). Patients with RA had significantly higher frequencies of comorbidities (*p* < 0.05), except for dementia, moderate or severe liver disease, and metastatic solid tumor (*p* > 0.05).

In univariate and multivariate analyses, RA was associated with HZ (Table 2). The incidence rate of HZ was higher in patients with RA than in the matched non-RA group (14.28 versus 7.36 events per 1000 person-years, adjusted IRR 1.74, 95% CI 1.65–1.84 with *p* <0.0001). We also found that RA increased HZ risk at the 1-year follow-up (adjusted IRR 30.42, 95% CI 18.02–51.36 with *p* < 0.0001). In the RA patient group, the RA with CIC RA matched with RA without CIC RA (adjusted IRR 1.54, 95% CI 1.35–1.77 with *p* < 0.0001); see Table 3.

For step two, Table 1 presents the demographic characteristics of the study population among patients with RA. We identified 1651 patients with CIC RA (mean age: 46.2 ± 12.9 years) and 18022 patients with non-CIC RA (mean age: 46.2 ± 13.6 years), from which we selected 11557 (1:7) matched patients (mean age: 46.2 ± 12.8 years). Patients with CIC RA had lower frequencies of lipid metabolism disorders, obesity, diabetes without and with chronic complications, hemiplegia, and paraplegia; patients with CIC RA had higher frequencies of chronic pulmonary disease, peptic ulcer disease, renal disease, and any malignancy comorbidities (*p* < 0.05). In univariate and multivariate analyses, CIC RA was associated with HZ (Table 3); the incidence rate of HZ was higher in patients with CIC RA than in matched patients with non-CIC RA (21.95 vs. 14.03 events per 1000 person-years, adjusted IRR 1.65, 95% CI 1.44–1.89). Moreover, CIC RA and the related incident HZ risk at the 1-year follow-up were analyzed (adjusted IRR 1.79, 95% CI 1.21–2.57; Table 3). CIC RA and the related incident HZ risk levels at the ≤1 year, >1 to 3 years, and >3 to 6 years of follow-up were analyzed (adjusted IRR 1.76, 1.73, 1.85, respectively; Table 3).

**Figure 2:**
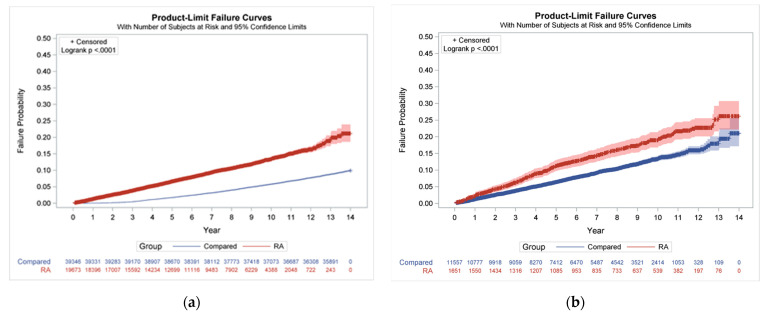
Cumulative incidence rates of herpes zoster. (**a**) Differences in the cumulative incidence rates of herpes zoster between patients with RA and comparison group. (**b**) Differences in the cumulative incidence rates of herpes zoster between RA patients with and without a CIC by using the log-rank test and Kaplan–Meier analysis. RA: rheumatoid arthritis, CIC: catastrophic illness certificate.

The cumulative incidence rates of herpes zoster of those with and those without RA, and those with RA with a CIC and without a CIC (using the log-rank test and Kaplan–Meier analysis), are significantly different, with log rank *p* < 0.0001 (Figure 2a,b). 

In Table 4 are the results of the control group and the RA patients for HZ complications (the adjusted hazard ratio (HR) was 2.85, 95% CI 2.53–3.21). For the RA groups, the HR of CIC RA associated with getting HZ complications (the adjusted HR was 1.78 95% CI 1.39–2.29), and both relations are statically significant at *p* < 0.0001. 

In step three, the associations of medications prescribed to CIC RA patients and HZ were estimated. Table 5 presents the HZ patients with medications used for CIC RA. Note that 27.4% of HZ patients had nervous-system complications. HZ incidence was significantly higher in women (86.5%; *p* = 0.0018) and in patients with prednisolone use (76.1%; *p* = 0.0095). We also tried to estimate prednisolone use’s association with the risk of HZ, but the difference was only marginally significant between HZ and non-HZ in non-CIC RA patients (*p* = 0.0874; Supplementary Tables S1 and S2).

To further evaluate the effect of prednisolone, use on HZ risk in patients with CIC RA, we classified the average prednisolone dose by stratifying the prednisolone exposure into yes or no and categorizing the total days and total dose (in mg) according to a quartile method. Synergistic effects of DMARDs or biopharmaceuticals with prednisolone were observed. Table 6 presents the relationship between prednisolone use or not and HZ risk (76.1% vs. 68.0%; adjusted OR 1.50, 95% CI 1.10–2.04). Patient prednisolone use analyzed by days and total dose revealed an increased HZ risk in the respective last quartiles (OR 2.25, 95% CI 1.54–3.30, and OR 2.04, 95% CI 1.39–3.00, respectively). The aforementioned results suggest a positive relationship between long-term prednisolone use (approximately 4.92 years (i.e., 1795 days)) and HZ risk, indicating that prednisolone was a major risk factor of HZ in patients with CIC RA. DMARDs combined with prednisolone showed an increased HZ risk compared with untreated patients (73.7% vs. 65.7%; OR 1.66, 95% CI 1.07–2.60 *p* = 0.0249), suggesting that prednisolone is often used in combination with DMARDs for treating RA in Taiwan. However, combining biopharmaceuticals with prednisolone did not significantly increase the risk of HZ (OR 1.31, 95% CI 0.81–2.12 *p* = 0.2740). Additionally, the biopharmaceuticals etanercept and adalimumab (anti-TNF-α agents) and rituximab (B-cell–depleting agent) had no significant association with HZ risk (Table 5 and Table 6)

## 4. Discussion

In this observational study, patients with RA had a 1.94-fold increased HZ risk compared with the matched non-RA group in Taiwan, which is consistent with a Japanese study (2.19-fold) showing an around two-times-higher chance of getting HZ for RA patients [28]. Among the RA group, patients with CIC RA had a 1.56-fold increase in HZ risk compared with patients with non-CIC RA. Notably, patients with RA during 1-year of follow-up were highly associated with HZ compared with the matched non-RA group (adjusted IRR 30.42, 95% CI 18.02–51.36 with *p* < 0.0001). Taken together, patients with RA were more likely to develop HZ (Table 2 and Table 3). Our results also showed that the nervous-system complications in CIC RA patients occurred 27.4% of the time with HZ infection, which might impair the quality of life of those patients. We also revealed that patients with CIC RA taking prednisolone had a dose-dependent increase in HZ risk (5 mg/day per year, OR 1.04, 1.01–1.07; Table 6). By contrast, the use of the biopharmaceuticals etanercept, adalimumab, and rituximab led to no significant increase in the risk of HZ in patients with CIC RA. HZ risk decreased with the use of biopharmaceutical agents only (the adjusted OR was 0.76 95% CI 0.17–3.39 *p* = 0.7217) when compared to using both biopharmaceutical agents and prednisolone. Then, with the use of only prednisolone (the adjusted OR was 1.50 95% CI 1.08–2.07 *p* = 0.0146) compared to combined treatment, the OR slightly dropped, though not significantly (the adjusted OR was 1.31 95% CI 0.81–2.12 *p* = 0.2740) (Table 6). The use of new treatments might reduce the hazard ratio of steroid use, but the numbers of patients on those new treatments is still very low compared to those on DMARDs and prednisolone, but we can pay more the attention to the new treatment outcomes in the further study.

Accumulating evidence from epidemiology studies and clinical trials has revealed that RA is associated with increased HZ risk [17,18,35]. Declining virus-specific cellular immunity to VZV infection occurs both naturally by aging and by immunosuppressive treatments, predisposing RA patients to an increased HZ risk within a few years of disease diagnosis [4,6]. In the prebiologic era, in addition to disease itself, several drugs used to treat RA, particularly corticosteroids, were implicated as potentially increasing the HZ risk [22]. Corticosteroids have been reported to increase the HZ risk not only in patients with RA, but also in patients with other autoimmune diseases, such as systemic lupus erythematosus [16,18,35,36]. In agreement with previous observations, our data also indicate that corticosteroids (prednisolone) increased the HZ risk in patients with CIC RA. Moreover, the increase in risk was dose-dependent: an approximately 4% to 6% increase in risk if the daily prednisolone dose was >5 mg in patients with CIC RA.

Among the DMARDs, hydroxychloroquine and MTX have been most frequently mentioned to increase the HZ risk [18,37]. In our data, we did not find a significant difference in the percentage of patients with CIC RA, with or without HZ, taking hydroxychloroquine (79.9% vs. 74.2%) or MTX (61.8% vs. 60.1%). In our study, DMARDs played a notable role: when used alone, they were not associated with an increased risk, but when combined with corticosteroids (prednisolone), they increased the HZ risk 1.67-fold compared with untreated patients (Table 5 and Table 6). This indicates synergistic effects of DMARDs with prednisolone. As DMARDs are the most indispensable drugs used for RA treatment and corticosteroids are often used in combination with DMARDs, the risk–benefit balance should be adjusted individually.

Since the introduction of biologics, the risks associated with TNF inhibitors have been widely discussed, but the data are conflicting [15,16,35,37,38,39]. A meta-analysis revealed a significantly increased risk of up to 61% [38]. By contrast, a large US multi-institutional collaboration study did not find an association between TNF inhibitor initiation and HZ risk [15]. Our data showed that TNF inhibitors (and rituximab, a B-cell-depleting agent) were not significantly associated with HZ risk in patients with CIC RA. Notably, biopharmaceuticals combined with prednisolone did not significantly increase the HZ risk compared with untreated patients. Therefore, low dose prednisolone and early taping of prednisolone need to be considered to reduce the complications. Our results showed that the risk of HZ was increased in CIC RA patients. Therefore, HZ vaccinations may be required in these patients to improve their life quality [39,40].

There are several limitations to our study. First, the disease activity of each patient could not be identified from the NHIRD. Disease activity affects the patient’s susceptibility to HZ, as discussed in previous reports [19,20,21,22]. Second, some of these patients may have sought treatment by using alternative medicines, which could have suppressed their immune function. For instance, triptolide is one such alternative medicine that is very often used to treat autoimmune diseases [41]. Finally, although we adjusted our data for age, sex, and comorbidities, some unknown confounding factors might still have impacted our conclusions.

## 5. Conclusions

Patients with RA are more likely to develop HZ early, particularly CIC RA patients at 1–year follow-up. We revealed that long-term use of corticosteroid (prednisolone; approximately 4.92 years) is strongly associated with HZ. Early tapering is therefore recommended to lower the risk of HZ. We found that in patients with CIC RA, the biopharmaceuticals had no significant association with HZ risk. Additionally, combining biopharmaceuticals with prednisolone did not significantly increase the risk of HZ. Our results might help physicians be alert to the possibility of early HZ in patients with RA, particularly in the first year of follow-up in patients with a daily prednisolone dose of >5 mg over 5 years. Our study might provide some important clinical messages to the physicians responsible for the decision making of RA patients’ treatment to reduce the risk of HZ.

## Figures and Tables

**Table 1:** Characteristics of patients with rheumatoid arthritis (RA) and RA with a catastrophic illness certificate (CIC).

	Step One			Step Two				
	RA	Matched Non-RA		RA				
			*p* Value	CIC	Non-CIC	*p* Value	1:7 Matched Non-CIC	*p* Value
N	19,673	39,346		1651	18,022		11,557	
Follow-up for HZ, median (IQR), years	4.1 (1.9–6.7)	9.1 (6.1–11.7)	<0.0001	3.9 (1.9–6.9)	4.2 (1.9–6.7)	0.9336	4.1 (1.9–6.7)	0.9070
Age of cohort entry mean (SD), years	46.2 (13.6)	46.0 (13.6)	0.0855	46.2 (12.9)	46.2 (13.6)	0.9522	46.2 (12.8)	0.9588
Age group, n (%)								
20 to 30	2401 (12.2)	4953 (12.6)		166 (10.1)	2235 (12.4)		1162 (10.1)	
>30 to 40	4544 (23.1)	9097 (23.1)		394 (23.9)	4150 (23.0)		2758 (23.9)	
>40 to 50	5459 (27.7)	11,088 (28.2)		487 (29.5)	4972 (27.6)		3409 (29.5)	
>50 to 60	3646 (18.5)	7146 (18.2)		330 (20.0)	3316 (18.4)		2310 (20.0)	
>60 to 70	2737 (13.9)	5316 (13.5)		221 (13.4)	2516 (14.0)		1547 (13.4)	
>70	886 (4.5)	1746 (4.4)	0.4047	53 (3.2)	833 (4.6)	0.0025	371 (3.2)	1.0000
Sex, females, n (%)	13,654 (69.4)	27,308 (69.4)	1.0000	1309 (79.3)	12,345 (68.5)	<0.0001	9163 (79.3)	1.0000
Region, n (%)								
Northern	9694 (49.3)	18,967 (48.2)		741 (44.9)	8953 (49.7)		5187 (44.9)	
Central	4420 (22.5)	8977 (22.8)		424 (25.7)	3996 (22.2)		2986 (25.8)	
Southern	4601 (23.4)	9304 (23.6)		420 (25.4)	4181 (23.2)		2945 (25.5)	
Eastern and other	820 (4.2)	1778 (4.5)		52 (3.1)	768 (4.3)		364 (3.1)	
Offshore islets	138 (0.7)	320 (0.8)	0.0414	14 (0.8)	124 (0.7)	<0.0001	75 (0.6)	0.9297
Comorbidities, n (%)								
Disorders of lipoid metabolism	7183 (36.5)	10,174 (25.9)	<0.0001	435 (26.3)	6748 (37.4)	<0.0001	4410 (38.2)	<0.0001
Obesity	264 (1.3)	311 (0.8)	<0.0001	14 (0.8)	250 (1.4)	0.0684	155 (1.3)	0.0953
Alcohol abuse	281 (1.4)	336 (0.9)	<0.0001	23 (1.4)	258 (1.4)	0.8996	145 (1.3)	0.6387
Hypertension	9169 (46.6)	15,010 (38.1)	<0.0001	746 (45.2)	8423 (46.7)	0.2261	5385 (46.6)	0.2824
Myocardial infarction	127 (0.6)	201 (0.5)	0.0380	10 (0.6)	117 (0.6)	0.8327	85 (0.7)	0.5594
Congestive heart failure	1089 (5.5)	1462 (3.7)	<0.0001	97 (5.9)	992 (5.5)	0.5282	788 (6.8)	0.1517
Peripheral vascular disease	850 (4.3)	1008 (2.6)	<0.0001	58 (3.5)	792 (4.4)	0.0917	623 (5.4)	0.0012
Cerebrovascular disease	1914 (9.7)	2874 (7.3)	<0.0001	144 (8.7)	1770 (9.8)	0.1491	1410 (12.2)	<0.0001
Dementia	237 (1.2)	470 (1.2)	0.9148	17 (1.0)	220 (1.2)	0.4958	174 (1.5)	0.1297
Chronic pulmonary disease	4373 (22.2)	5650 (14.4)	<0.0001	404 (24.5)	3969 (22.0)	0.0221	3196 (27.7)	0.0066
Peptic ulcer disease	6224 (31.6)	7477 (19.0)	<0.0001	591 (35.8)	5633 (31.3)	0.0001	4574 (39.6)	0.0032
Mild liver disease	4083 (20.8)	4688 (11.9)	<0.0001	333 (20.2)	3750 (20.8)	0.5405	3000 (26)	<0.0001
Moderate or severe liver disease	33 (0.2)	49 (0.1)	0.1840	7 (0.4)	26 (0.1)	0.0079	20 (0.2)	0.0347
Diabetes (without chronic complication)	2778 (14.1)	4250 (10.8)	<0.0001	189 (11.4)	2589 (14.4)	0.0011	2071 (17.9)	<0.0001
Diabetes (with chronic complication)	834 (4.2)	1284 (3.3)	<0.0001	57 (3.5)	777 (4.3)	0.0973	622 (5.4)	0.0009
Hemiplegia or paraplegia	381 (1.9)	449 (1.1)	<0.0001	21 (1.3)	360 (2.0)	0.0406	298 (2.6)	0.0012
Renal disease	789 (4.0)	891 (2.3)	<0.0001	87 (5.3)	702 (3.9)	0.0064	529 (4.6)	0.2121
Any malignancy	1053 (5.4)	1642 (4.2)	<0.0001	157 (9.5)	896 (5.0)	<0.0001	733 (6.3)	<0.0001
Metastatic solid tumor	53 (0.3)	76 (0.2)	0.0615	10 (0.6)	43 (0.2)	0.0059	31 (0.3)	0.0211

IQR: interquartile range; SD: standard deviation. Comorbidities were defined by more than three outpatient claims. Data of continuous and categorical variables were analyzed using the *t*-test or Wilcoxon rank sum test and chi-squared test to compare the data of rheumatoid arthritis with and without a CIC.

**Table 2:** Early risk of incident herpes zoster (HZ) in patients with rheumatoid arthritis (RA).

	Herpes Zoster/ Total Patients, %	Person-Years	Events per 1000 Person-Years (95% CI)	IRR (95% CI)	*p* value	Adjusted IRR (95% CI)	*p* Value
Follow-up ≤1 year							
Compared group	15/39,346, 0.04	39,339.10	0.38 (0.38–0.39)	1.00		1.00	
Rheumatoid arthritis	246/19,673, 1.25	19,072.90	12.90 (12.72–13.08)	33.83 (20.09–56.97)	<0.0001	30.42 (18.02–51.36)	<0.0001
Follow-up >1 to 3 years *							
Compared group	163/39,331, 0.41	117,874.45	1.38 (1.37–1.39)	1.00		1.00	
Rheumatoid arthritis	445/18,386, 2.42	52,406.54	8.49 (8.42–8.56)	6.14 (5.13–7.35)	<0.0001	5.61 (4.67–6.73)	<0.0001
Follow-up >3 to 6 years *							
Compared group	777/39,168, 1.98	233,851.40	3.32 (3.31–3.34)	1.00		1.00	
Rheumatoid arthritis	588/15,582, 3.77	87,023.67	6.76 (6.71–6.80)	2.03 (1.83–2.26)	<0.0001	1.80 (1.61–2.01)	<0.0001
Overall							
Compared group	3903/39,346, 9.92	530,337.81	7.36 (7.34–7.38)	1.00		1.00	
Rheumatoid arthritis	1857/19,673, 9.44	130,009.31	14.28 (14.21–14.36)	1.94 (1.84–2.05)	<0.0001	1.74 (1.65–1.84)	<0.0001

Incidence rate ratio (IRR) was calculated by using a generalized linear model to perform Poisson regression analysis (a log-linear model). Adjusted IRR was calculated after adjustment for region and significant comorbidities of disorders of lipoid metabolism, obesity, alcohol abuse, hypertension, myocardial infarction, congestive heart failure, peripheral vascular disease, cerebrovascular disease, chronic pulmonary disease, peptic ulcer disease, mild liver disease, diabetes (without chronic complication), diabetes (with chronic complication), renal disease, hemiplegia or paraplegia, and any malignancy. * For follow-up analysis, we calculated RA and risk of incident HZ at the 1-year follow-up; at the 3-year follow-up, excluding patients of <1 year of follow-up; 6-year follow-up, excluding patients of ≤3 years of follow-up; and >6-year follow-up, excluding patients with ≤6 years of follow-up.

**Table 3:** Association of rheumatoid arthritis (RA) with a catastrophic illness certificate (CIC) and herpes zoster (HZ).

	Herpes Zoster/ Total Patients, %	Person-Years	Events per 1000 Person-Years (95% CI)	IRR (95% CI)	*p* Value	Adjusted IRR (95% CI)	*p* Value
Follow-up ≤1 year							
RA without CIC	142/11,557, 1.23	11,191.86	12.69 (12.45–12.93)	1.00		1.00	
RA with CIC	34/1651, 2.06	1604.88	21.19 (20.17–22.25)	1.67 (1.15–2.43)	0.0072	1.76 (1.21–2.57)	0.0033
Follow-up >1 to 3 years *							
RA without CIC	256/10,773, 2.38	30,604.27	8.36 (8.27–8.46)	1.00		1.00	
RA with CIC	62/1550, 4.00	4427.16	14.00 (13.60–14.42)	1.67 (1.27–2.21)	0.0003	1.73 (1.31–2.29)	0.0001
Follow-up >3 to 6 years *							
RA without CIC	336/9052, 3.71	50,588.11	6.64 (6.58–6.70)	1.00		1.00	
RA with CIC	84/1316, 6.38	7376.68	11.39 (11.13–11.65)	1.71 (1.35–2.18)	<0.0001	1.85 (1.45–2.35)	<0.0001
Overall							
RA without CIC	1055/11,557, 9.13	75,184.83	14.03 (13.93–14.13)	1.00		1.00	
RA with CIC	259/1651, 15.69	11,798.50	21.95 (21.56–22.35)	1.56 (1.37–1.79)	<0.0001	1.65 (1.44–1.89)	<0.0001

Incidence rate ratio (IRR) was calculated by using a generalized linear model to perform Poisson regression analysis (a log-linear model). Adjusted IRR was calculated after adjustment for significant comorbidities of disorders of lipoid metabolism, peripheral vascular disease, cerebrovascular disease, chronic pulmonary disease, peptic ulcer disease, mild liver disease, moderate or severe liver disease, diabetes (without chronic complication), diabetes (with chronic complication), hemiplegia or paraplegia, any malignancy, and metastatic solid tumor. * For follow-up analysis, we calculated RA and risk of incident HZ at the 1-year follow-up; at the 3-year follow-up, excluding patients of <1 year of follow-up; 6-year follow-up, excluding patients of ≤3 years of follow-up; and >6-year follow-up, excluding patients with ≤6 years of follow-up.

**Table 4:** Association of rheumatoid arthritis (RA) with or without a catastrophic illness certificate (CIC) and herpes zoster complications.

	Herpes Zoster Complications, n (%)	Herpes Zoster without Complication, n(%)	Total Patients	Herpes Zoster Complications				Herpes Zoster without Complication			
				Crude HR (95% CI)	*p* Value	Adjusted HR (95% CI)	*p* Value	Crude HR (95% CI)	*p* Value	Adjusted HR (95% CI)	*p* Value
Compared group	952 (2.42)	2951 (7.50)	39,346	1.00		1.00		1.00		1.00	
RA Patients	550 (2.80)	1307 (6.64)	19,673	3.33 (2.96–3.74)	<0.0001	2.85 (2.53–3.21)	<0.0001	2.33 (2.17–2.49)	<0.0001	2.11 (1.96–2.26)	<0.0001
RA group											
RA without CIC	310 (2.68)	745 (6.45)	11,557	1.00		1.00		1.00		1.00	
RA with CIC	80 (4.85)	179 (10.84)	1651	1.64 (1.28–2.10)	<0.0001	1.78 (1.39–2.29)	<0.0001	1.55 (1.31–1.82)	<0.0001	1.61 (1.37–1.90)	<0.0001

Hazard ratio (HR) was calculated by using a Cox proportional hazards regression model. Adjusted HR was calculated after adjustment for significant comorbidities of disorders of lipoid metabolism, peripheral vascular disease, cerebrovascular disease, chronic pulmonary disease, peptic ulcer disease, mild liver disease, moderate or severe liver disease, diabetes (without chronic complication), diabetes (with chronic complication), hemiplegia or paraplegia, any malignancy, and metastatic solid tumor. Herpes zoster complications: meningitis (ICD-9-CM 053.0), nervous system complications (053.1), or ophthalmic complications (053.2).

**Table 5:** HZ association with medications used by CIC RA patients.

	Herpes Zoster	Non-Herpes Zoster	*p* Value
N	259	1392	
Herpes zoster complications			
Meningitis (ICD-9-CM 053.0), n (%)	2 (0.8)		
Nervous system complications (053.1), n (%)	71 (27.4)		
Ophthalmic complications (053.2), n (%)	8 (3.1)		
Age of cohort entry mean (SD), years	50.0 (12.1)	45.5 (12.9)	<0.0001
Age group, n (%)			
20 to 30	12 (4.6)	154 (11.1)	
>30 to 40	41 (15.8)	353 (25.4)	
>40 to 50	78 (30.1)	409 (29.4)	
>50 to 60	69 (26.6)	261 (18.8)	
>60 to 70	48 (18.5)	173 (12.4)	
>70	11 (4.2)	42 (3.0)	<0.0001
Sex, females, n (%)	224 (86.5)	1085 (78.0)	0.0018
Region, n (%)			
Northern	124 (47.9)	617 (44.3)	
Central	74 (28.6)	350 (25.1)	
Southern	48 (18.5)	372 (26.7)	
Eastern and other	10 (3.9)	42 (3.0)	
Offshore islets	3 (1.2)	11 (0.8)	0.0840
Comorbidities, n (%)			
Disorders of lipoid metabolism	73 (28.2)	362 (26.0)	0.4647
Obesity	3 (1.2)	11 (0.8)	0.5531
Alcohol abuse	2 (0.8)	21 (1.5)	0.3532
Hypertension	134 (51.7)	612 (44.0)	0.0210
Myocardial infarction	2 (0.8)	8 (0.6)	0.7068
Congestive heart failure	20 (7.7)	77 (5.5)	0.1687
Peripheral vascular disease	7 (2.7)	51 (3.7)	0.4405
Cerebrovascular disease	22 (8.5)	122 (8.8)	0.8875
Dementia	5 (1.9)	12 (0.9)	0.1178
Chronic pulmonary disease	79 (30.5)	325 (23.3)	0.0139
Peptic ulcer disease	99 (38.2)	492 (35.3)	0.3748
Mild liver disease	56 (21.6)	277 (19.9)	0.5259
Diabetes (without chronic complication)	32 (12.4)	157 (11.3)	0.6173
Diabetes (with chronic complication)	9 (3.5)	48 (3.4)	0.9828
Hemiplegia or paraplegia	6 (2.3)	15 (1.1)	0.1023
Renal disease	18 (6.9)	69 (5.0)	0.1875
Any malignancy	27 (10.4)	130 (9.3)	0.5845
Moderate or severe liver disease	2 (0.8)	5 (0.4)	0.3476
Metastatic solid tumor	2 (0.8)	8 (0.6)	0.7068
Medications use (ATC code), n (%)			
Corticosteroid use			
Prednisolone (H02AB06)	197 (76.1)	946 (68.0)	0.0095
Methylprednisolone (H02AB04)	32 (12.4)	177 (12.7)	0.8728
Dexamethasone (H02AB02)	63 (24.3)	266 (19.1)	0.0537
Biopharmaceutical			
Etanercept (L04AB01)	24 (9.3)	130 (9.3)	0.9705
Adalimumab (L04AB04)	11 (4.2)	58 (4.2)	0.9526
Rituximab (L01XC02)	2 (0.8)	11 (0.8)	0.9760
Combined biopharmaceutical use	32 (12.4)	180 (12.9)	0.7992
Disease-modifying antirheumatic drugs (DMARDs)			
Azathioprine (L04AX01)	17 (6.6)	82 (5.9)	0.6753
Methotrexate (L01BA01)	160 (61.8)	836 (60.1)	0.6037
Sulfasalazine (A07EC01)	170 (65.6)	866 (62.2)	0.2952
Hydroxychloroquine (P01BA02)	207 (79.9)	1033 (74.2)	0.0509
Leflunomide (L04AA13)	40 (15.4)	268 (19.3)	0.1485
Ciclosporin (L04AA01)	37 (14.3)	185 (13.3)	0.6663
Combined DMARDs use	227 (87.6)	1154 (82.9)	0.0581
Other medications			
Cyclophosphamide (L01AA01)	9 (3.5)	28 (2.0)	0.1440
Penicillamine (M01CC01)	14 (5.4)	69 (5.0)	0.7616

SD: standard deviation; DMARDs: disease-modifying antirheumatic drugs; ATC code: Anatomical Therapeutic Chemical code; CIC RA: catastrophic illness-certified rheumatoid arthritis. Data of continuous and categorical variables were analyzed using *t*-tests and chi-squared tests to compare the data of herpes zoster (HZ) and the non-HZ group.

**Table 6:** Prednisolone, biopharmaceutical, and DMARD use associated with HZ risk in CIC RA patients.

	HZ	Non-HZ	OR (95% CI)	*p*-Value	Adjusted OR (95% CI)	*p*-Value
N	259	1392				
Model 1: Prednisolone (H02AB06), n (%)						
No	62 (23.9)	446 (32.0)	1.00		1.00	
Yes	197 (76.1)	946 (68.0)	1.50 (1.10–2.04)	0.0099	1.48 (1.08–2.03)	0.0140
Prednisolone use (days), n (%)						
No use	62 (23.9)	446 (32.0)	1.00		1.00	
<172	43 (16.6)	243 (17.5)	1.27 (0.84–1.94)	0.2592	1.31 (0.85–2.00)	0.2189
173–668	48 (18.5)	238 (17.1)	1.45 (0.96–2.18)	0.0741	1.51 (0.99–2.29)	0.0520
669–1795	38 (14.7)	248 (17.8)	1.10 (0.72–1.7)	0.6592	1.05 (0.68–1.63)	0.8340
>1795	68 (26.3)	217 (15.6)	2.25 (1.54–3.3)	<0.0001	2.16 (1.46–3.19)	0.0001
Per 1 year *			1.07 (1.02–1.12)	0.0026	1.06 (1.02–1.12)	0.0098
Prednisolone use (dosages, mg), n (%)						
No use	62 (23.9)	446 (32.0)	1.00		1.00	
<1050	43 (16.6)	245 (17.6)	1.26 (0.83–1.92)	0.2755	1.29 (0.84–1.98)	0.2375
1051–3990	45 (17.4)	239 (17.2)	1.35 (0.89–2.05)	0.1517	1.39 (0.91–2.11)	0.1302
3990–10995	46 (17.8)	240 (17.2)	1.38 (0.91–2.08)	0.1269	1.33 (0.87–2.02)	0.1864
>10995	63 (24.3)	222 (15.9)	2.04 (1.39–3.00)	0.0003	1.95 (1.31–2.89)	0.0009
Per 1825 mg/year			1.04 (1.01–1.07)	0.0097	1.04 (1.01–1.07)	0.0156
Model 2: Combined biopharmaceutical and prednisolone						
No/No	60 (23.2)	427 (30.7)	1.00		1.00	
Yes/No	2 (0.8)	19 (1.4)	0.75 (0.17–3.30)	0.7024	0.76 (0.17–3.39)	0.7217
No/Yes	167 (64.5)	785 (56.4)	1.51 (1.10–2.08)	0.0105	1.50 (1.08–2.07)	0.0146
Yes/Yes	30 (11.6)	161 (11.6)	1.33 (0.83–2.13)	0.2435	1.31 (0.81–2.12)	0.2740
Model 3: Combined DMARDs and prednisolone						
No/No	26 (10.0)	206 (14.8)	1.00		1.00	
Yes/No	36 (13.9)	240 (17.2)	1.19 (0.69–2.03)	0.5291	1.23 (0.71–2.13)	0.4638
No/Yes	6 (2.3)	32 (2.3)	1.49 (0.57–3.89)	0.4203	1.51 (0.57–4.02)	0.4122
Yes/Yes	191 (73.7)	914 (65.7)	1.66 (1.07–2.56)	0.0236	1.67 (1.07–2.60)	0.0249

DMARDs: disease-modifying antirheumatic drugs. We classified the average prednisolone dose by using three approaches: stratifying the prednisolone exposure into yes or no and categorizing the total days and total dosage (mg) according to a quartile method. Adjusted odds ratio (OR) was calculated after adjustment for age group, sex, and comorbidities by using a multiple logistic regression model. * Per 1825 mg/year was derived as follows: 5 mg/days × 365 days.

## Data Availability

Not applicable.

## Supplementary Materials

The following supporting information can be downloaded at: https://www.mdpi.com/article/10.3390/ijerph20032123/s1. Supplementary Table S1. Characteristics of rheumatoid arthritis patients with a non-catastrophic illness certificate (non-CIC) with or without herpes zoster. Supplementary Table S2. Prednisolone use associated with HZ risk in non-CIC RA patients.
