# Supplementary material for: Herpes Zoster Risk in Patients with Rheumatoid Arthritis and Its Association with Medications Used
Source: Int J Environ Res Public Health. 2023 Jan 24;20(3):2123. doi: 10.3390/ijerph20032123 (PMC9915285; doi:10.3390/ijerph20032123)
Supplement: Supplementary file 1 [file ijerph-20-02123-s001.zip › ijerph-2028062-supplementary.pdf]

International Journal of *Environmental Research and Public Health*

MDPI

Supplementary Table S1. Characteristics of rheumatoid arthritis patients with a non-catastrophic illness certificate (non-CIC) with or without herpes zoster.

	Rheumatoid arthritis with a non-catastrophic illness certificate (non-CIC)		
	Herpes zoster	Non-herpes zoster	P value
N	1055	10502	
Herpes zoster complications			
Meningitis (ICD-9-CM 053.0), n(%)	13 (1.2)		
Nervous system complications (053.1), n(%)	270 (25.6)		
Ophthalmic complications (053.2), n(%)	41 (3.9)		
Age of cohort entry mean (SD), years	49.96 (12.02)	45.82 (12.86)	<0.0001
Age group, n (%)			
20 to 30	41 (3.9)	1121 (10.7)	
>30 to 40	167 (15.8)	2591 (24.7)	
>40 to 50	354 (33.6)	3055 (29.1)	
>50 to 60	265 (25.1)	2045 (19.5)	
>60 to 70	181 (17.2)	1366 (13.0)	
>70	47 (4.5)	324 (3.1)	<0.0001
Sex, females, n(%)	845 (80.1)	8318 (79.2)	0.2964
Region, n (%)			
Northern	481 (45.6)	4706 (44.8)	0.3970
Central	284 (26.9)	2702 (25.7)	0.0005
Southern	243 (23.0)	2702 (25.7)	0.0143
Eastern and other	40 (3.8)	324 (3.1)	0.0020
Offshore islets	7 (0.7)	68 (0.6)	0.1048
Comorbidities, n (%)			
Disorders of lipoid metabolism	495 (46.9)	3915 (37.3)	<0.0001
Obesity	7 (0.7)	148 (1.4)	0.0447
Alcohol abuse	5 (0.5)	140 (1.3)	0.0169
Hypertension	574 (54.4)	4811 (45.8)	<0.0001
Myocardial infarction	10 (0.9)	75 (0.7)	0.3970
Congestive heart failure	99 (9.4)	689 (6.6)	0.0005
Peripheral vascular disease	74 (7.0)	549 (5.2)	0.0143
Cerebrovascular disease	160 (15.2)	1250 (11.9)	0.0020
Dementia	22 (2.1)	152 (1.4)	0.1048
Chronic pulmonary disease	377 (35.7)	2819 (26.8)	<0.0001
Peptic ulcer disease	491 (46.5)	4083 (38.9)	<0.0001
Mild liver disease	295 (28.0)	2705 (25.8)	0.1194
Diabetes (without chronic complication)	2 (0.2)	18 (0.2)	0.8923
Diabetes (with chronic complication)	242 (22.9)	1829 (17.4)	<0.0001
Hemiplegia or paraplegia	83 (7.9)	539 (5.1)	0.0002
Renal disease	42 (4.0)	256 (2.4)	0.0026
Any malignancy	77 (7.3)	452 (4.3)	<0.0001
Moderate or severe liver disease	80 (7.6)	653 (6.2)	0.0829
Metastatic solid tumor	3 (0.3)	28 (0.3)	0.9154
Medications use (ATC code), n(%)			
Corticosteroid use			
Prednisolone (H02AB06)	99(9.4)	828(7.9)	0.0874
Methylprednisolone (H02AB04)	1(0.1)	24(0.2)	0.3728
Dexamethasone (H02AB02)	35(3.3)	279(2.7)	0.2081
Biopharmaceutical			
Etanercept (L04AB01)	0 (0.0)	0 (0.0)	1.0000
Adalimumab (L04AB04)	0 (0.0)	1 (0.0)	1.0000
Rituximab (L01XC02)	0 (0.0)	0 (0.0)	1.0000

Combined biopharmaceutical use	0 (0.0)	1 (0.0)	1.0000
Disease-modifying antirheumatic drugs (DMARDs)			
Azathioprine (L04AX01)	0 (0.0)	8 (0.1)	0.3698
Methotrexate (L01BA01)	25 (2.4)	277 (2.6)	0.6030
Sulfasalazine (A07EC01)	55 (5.2)	562 (5.4)	0.8492
Hydroxychloroquine (P01BA02)	81 (7.7)	770 (7.3)	0.6819
Leflunomide (L04AA13)	2 (0.2)	15 (0.1)	0.7057
Ciclosporin (L04AA01)	0 (0.0)	10 (0.1)	0.3160
Combined DMARDs use	114 (10.8)	1147 (10.9)	0.9083
Other			
Cyclophosphamide (L01AA01)	0 (0.0)	0 (0.0)	1.0000
Penicillamine (M01CC01)	1(0.1)	9(0.1)	0.9238

IQR: interquartile range; SD: standard deviation.
Comorbidities were defined as more than three outpatient claims.
Data of continuous and categorical variables were analyzed using the *t*-test and chi-squared test to compare the data of the herpes zoster group and the non-herpes zoster group.
ATC code: Anatomical Therapeutic Chemical code.

Supplementary Table S2. Prednisolone use associated with HZ risk in non-CIC RA patients.

	HZ	Non-HZ	OR (95% CI)	P-value	Adjusted OR (95% CI)	P-value
N	1055	10502				
Model 1: Prednisolone (H02AB06), n(%)						
No	956 (90.6)	9674 (92.1)	1.00		1.00	
Yes	99 (9.4)	828 (7.9)	1.21 (0.97-1.51)	0.0877	1.21 (0.97-1.51)	0.0635
Prednisolone use (days), n(%)						
No use	956 (90.6)	9674 (92.1)	1.00		1.00	
<172	88 (8.3)	729 (6.9)	1.22 (0.97-1.54)	0.0895	1.22 (0.97-1.54)	0.0662
173-668	8 (0.8)	67 (0.6)	1.21 (0.58-2.52)	0.6145	1.21 (0.58-2.52)	0.5366
669-1795	3 (0.3)	25 (0.2)	1.21 (0.37-4.03)	0.7510	1.21 (0.37-4.03)	0.8149
>1795	0 (0.0)	7 (0.1)	-	-	-	-
Per 1 year*			0.97 (0.72-1.30)	0.8161	0.97 (0.72-1.30)	0.7370
Prednisolone use (dosages, mg), n(%)						
No use	956 (90.6)	9674 (92.1)	1.00		1.00	
<1050	90 (8.5)	733 (7.0)	1.24 (0.99-1.56)	0.0629	1.24 (0.99-1.56)	0.0446
1051-3990	6 (0.6)	69 (0.7)	0.88 (0.38-2.03)	0.7645	0.88 (0.38-2.03)	0.8181
3990-10995	3 (0.3)	20 (0.2)	1.52 (0.45-5.12)	0.5009	1.52 (0.45-5.12)	0.4996
>10995	0 (0.0)	6 (0.1)	-	-	-	-
Per 1825 mg/year			0.94 (0.72-1.23)	0.6498	0.94 (0.72-1.23)	0.5788
Model 4: Combined biopharmaceutical and prednisolone						
No/No	956 (90.6)	9673 (92.1)	1.00		1.00	
Yes/No	0 (0.0)	1 (0.0)	-	-	-	-
No/Yes	99 (9.4)	828 (7.9)	1.21 (0.97-1.51)	0.0880	1.21 (0.97-1.51)	0.0636
Yes/Yes	0 (0.0)	0 (0.0)	-	-	-	-
Model 3: Combined DMARDs and prednisolone						
No/No	890(84.4)	8973 (85.4)	1.00		1.00	
Yes/No	66 (6.3)	701 (6.7)	0.95 (0.73-1.23)	0.6963	0.95 (0.73-1.23)	0.9079
No/Yes	51 (4.8)	382 (3.6)	1.35 (0.99-1.82)	0.0520	1.35 (1.00-1.82)	0.0373
Yes/Yes	48 (4.5)	446 (4.2)	1.09 (0.8-1.47)	0.6006	1.09 (0.80-1.47)	0.5101

DMARDs: disease-modifying antirheumatic drugs.
We classified the average prednisolone dose by using three approaches: stratifying the prednisolone exposure into yes or no and categorizing the total days and total dosage (mg) according to a quartile method.
All variables in **Supplementary** Table 2 with a *p*-value of less than 0.1 in the univariate model were further entered into the multivariate analysis.

Adjusted odds ratio (OR) was calculated after adjustment for age group, sex, and comorbidities by using a multiple logistic regression model.
*Per 1825 mg/year was derived as follows: 5 mg/days × 365 days.
